# Visual function and disability are associated with microcystic macular edema, macular and peripapillary vessel density in patients with neuromyelitis optica spectrum disorder

**DOI:** 10.3389/fneur.2022.1019959

**Published:** 2022-11-14

**Authors:** Jin Li, Yihong Chen, Ying Zhang, Zhiyong He, Huankai Yu, Ce Shi, Meixiao Shen, Fan Lu

**Affiliations:** ^1^Oujiang Laboratory (Zhejiang Lab for Regenerative Medicine, Vision and Brain Health), Wenzhou, China; ^2^School of Ophthalmology and Optometry, Wenzhou Medical University, Wenzhou, China; ^3^Department of Ophthalmology, Affiliated Hangzhou First People's Hospital, Zhejiang University School of Medicine, Hangzhou, China; ^4^The Second Affiliated Hospital and Yuying Children's Hospital of Wenzhou Medical University, Wenzhou, China

**Keywords:** neuromyelitis optica spectrum disorder, microcystic macular edema, optical coherence tomography angiography, visual impairment, disability

## Abstract

**Purpose:**

To assess macular and peripapillary vessel density and neurodegeneration in eyes with and without microcystic macular edema (MME) in neuromyelitis optica spectrum disorder (NMOSD) patients while investigating their association with visual impairment and disease disability.

**Methods:**

This is a cross-sectional study. A total of 52 eyes from 29 NMOSD patients were recruited, including 8 eyes with MME from 7 patients. Optical coherence tomography angiography (OCTA) images were analyzed to quantify the radial papillary capillary density (RPCD), and the density of macular microvascular network in both the superficial retinal capillary plexus (SRCP) and the deep retinal capillary plexus (DRCP). Thicknesses of the neural retinal layers centered on the fovea and the optic nerve head were also collected by OCT. Best-corrected visual acuity (BCVA) and Expanded Disability Status Scale (EDSS) scores were assessed for all patients. Microvascular densities and retinal sublayer thicknesses were compared among groups, and correlations of these vascular and structural parameters with BCVA and EDSS scores were determined.

**Results:**

Patients with NMOSD and MME had significantly decreased visual acuity and worse EDSS score than patients without MME (*P* = 0.01 and 0.002, respectively). The vessel density in SRCP and RPCD were significantly lower in eyes with MME and ON compared to that of eyes with ON but without MME and eyes without MME or ON. Impairment of visual acuity and disease severity were significantly negatively associated with the reduction of SRCP vessel density and RPCD but were not related to DRCP vessel density.

**Conclusions:**

MME were correlated with worse visual impairment and disability in NMOSD patients. Sparse SRCP vessel density and RPCD were observed in NMOSD MME eyes and correlated with worse BCVA and EDSS scores.

## Introduction

Neuromyelitis optica spectrum disorder (NMOSD) is an inflammatory disease of the central nervous system) and is characterized by injury to the optic nerve, spinal cord, brainstem, and/or brain parenchyma, which can cause severe visual dysfunction and ambulatory disability (1, 2). The retina is one of the sites of inflammation as a result of blood-retinal barrier disruption in NMOSD. Sometimes observed in NMOSD, microcystic macular edema (MME) is a retinal phenotype characterized by honeycombed, microcystic abnormalities of the inner nuclear layer (INL) on optical coherence tomography (OCT). It is observed to be the result of many potential causes, such as glaucoma, uveitis, retinal vein occlusion, and age-related macular degeneration (3–5). Recent evidence suggests that MME occurs in about 20–26% of NMOSD patients and 5–6% of MS patients (6, 7). While the exact pathogenesis of MME in NMOSD remains unclear, findings in several studies suggest it could be related to the retinal vascular system, as there is evidence that NMOSD patients have altered retinal microvasculature (8, 9). While using OCT, Brar et al. reported that microcysts are found within the inner nuclear layer and demonstrate diffuse fluorescein leakage which is a marker of blood-retinal barrier breakdown (10). This suggests that an abnormal hemodynamic status may play a part in the development of MME.

Optical coherence tomography angiography (OCTA) is a non-invasive procedure used for the analysis of retinal perfusion qualitatively and quantitatively with high resolution. Moreover, this technique provides in-depth information by way of showing the retinal microvascular network in different retinal layers (11, 12). With the emergence of OCTA, it is feasible to examine the correlation between microvascular perfusion and the development of MME. Thus, the goals of the current study were to characterize the retinal microvascular network in patients with NMOSD and MME and then to investigate the relationship of microvascular perfusion and visual impairment or disease disability.

## Methods

### Subjects

Patients with NMOSD were enrolled from the neurology clinic of the Second Affiliated Hospital, Wenzhou Medical University, Wenzhou, China. The patients enrolled were diagnosed with NMOSD by two experienced neurologists according to the 2015 criteria of the International Panel for NMO Diagnosis. Serum AQP4-ab were evaluated with a fixed cell-based indirect immunofluorescence test at a branch of the EUROIMMUN Medical Diagnostic Laboratory in China (EUROIMMUN AG, Lübeck, Germany). Expanded Disability Status Scale (EDSS) scores were recorded after a clinical neurological examination. The relapse preventative regimens for the patients were mycophenolate mofetil (*N* = 16), azathioprine (*N* = 4), rituximab (*N* = 2), and intermittent intravenous immunoglobulin (*N* = 1). The remaining 6 patients were not receiving any preventive medications at the time of inclusion.

All of the patients underwent a complete ophthalmological examination, including refraction and best corrected visual acuity (BCVA) examination, axial length (AL) measurement, intraocular pressure (IOP) measurement, slit-lamp biomicroscopy, funduscopic examination, OCT, and OCTA imaging. Inclusion criteria were as follows: refractive errors within +3.00 diopters (D) to −5.00 D of spherical equivalent (SE), BCVA ≥ 20/400, IOP <21 mm Hg, eyes without significant media opacities, no ON attack within the previous 6 months before enrolment, and serum AQP4-ab positive. Patients with any diagnosis of other ophthalmologic diseases (especially diseases that may lead to retinal edema such as uveitis or diabetic retinopathy) or underwent previous ocular surgery were excluded.

The diagnosis of optic neuritis (ON) was based on the presence of acute progression of vision loss associated with pain on eye movement, color vision and/or visual field impairments, and a compatible optic nerve enhancement visualized by MRI (13). History of ON attack was recorded according to medical records. Orbital MRI with T2-weighted imaging and gadolinium-enhanced T1 sequences were examined to verify the ON involved eye.

MME was evaluated using OCT images by two ophthalmologists independently and was defined as a band of perifoveal, thin, elongated ‘cysts' of hypo-reflectivity with clear boundaries in the INL evident on at least 2 contiguous B-scans, or visible in a comparable region on at least 2 separate B-scans (7). Two readers agreed in all cases of MME.

The study adhered to the tenets of the Declaration of Helsinki and was approved by the Ethics Committee of the Eye Hospital of Wenzhou Medical University, Wenzhou, China. Written informed consent was obtained from all individuals enrolled in this study.

### SD-OCT procedure

All eyes were imaged with an SD-OCT system (Optovue RTVue XR Avanti; Optovue, Inc., Fremont, California, USA; software V. 2017.1.0.155) using an 840 nm wavelength laser with a scan speed of 70,000 A-scans per second. The radial scan pattern (8-mm diameter; 18 lines), centered on the fovea, was performed to evaluate the existence of MME as well as other abnormalities of the retina or vitreoretinal interface. Automatic segmentation was conducted by a custom software program based on the gradient information and shortest path search that was developed in MATLAB for OCT image analysis as described in previous studies (14, 15). Images were manually confirmed through visual inspection by a masked grader after automatic segmentation. Macular retinal nerve fiber layer (mRNFL) and ganglion cell layer plus inner plexiform layer (GCL-IPL) thicknesses were obtained. The macular thickness map was divided into 9 sections, including a central (1 mm diameter), inner ring (1–3 mm diameter), and external ring (3–6 mm diameter). Each ring was divided into 4 quadrants: superior region (S1, S2), nasal region (N1, N2), inferior region (I1, I2), and temporal region (T1, T2). The peripapillary retinal nerve fiber layer (pRNFL) thickness was acquired by the HD Angio Disc 4.5 mm pattern which provides thickness metrics of a 2–4 mm ring area around the optic disc. Only scans with high quality of scan quality (SQ) ≥ 6 were selected for further analysis.

### OCT angiography

OCT angiography images were acquired using Angio Vue software 2.0 of the Avanti RTVue-XR, which detected erythrocyte movement basing on split-spectrum-amplitude-decorrelation angiography (SSADA) algorithm. The macular microcapillaries around the foveal center were acquired by the AngioRetina mode (3 × 3 mm macular cube), and the superficial retinal capillary plexus (SRCP) and deep retinal capillary plexus (DRCP) vessel densities were automatically generated by the software, corresponding to the slab extending from the internal limiting membrane (ILM) to 9 μm above the IPL to 9 μm below the outer plexiform layer. The HD Angio Disc 4.5 mm protocol automatically provides not only pRNFL thickness, but also the radial peripapillary capillary density (RPCD) within the ILM, as well as the nerve fiber layer within a 2–4 mm annular zone around the optic disc. Vessel densities, defined as the percentage of area occupied by OCTA detected vasculature, were automatically computed and generated. Poor quality images with a SQ <6 or with residual motion artifacts were excluded.

### Statistical analysis

The distribution type of all the continuous variables were assessed by the Shapiro-Wilk test. Normal distributed descriptive results were presented as the mean ± standard deviation whereas non-normal distributed variables were presented as the median. χ^2^ test were used for categorical variables. All eyes were divided into three groups: (1) NMOSD non-MME non-ON group: eyes of NMOSD patients without MME or ON; (2) NMOSD non-MME ON group: eyes of NMOSD patients without MME but with ON; (3) NMOSD MME ON group: eyes of NMOSD patients with MME and ON. To maximize the use of available data, two eyes of one subject were included in the statistical analysis. The generalized estimating equations (GEE) model was utilized to decrease the impact of possible correlation of observations from the same subjects. The correlation between BCVA/EDSS and OCT (A) parameters was analyzed using the GEE models. Data analysis was performed using IBM SPSS software (Ver.22) and MedCalc V.10.1.3.0 f (MedCalc Software, Ostend, 124 Belgium, www.medcalc.be). A *P* < 0.05 was considered to be statistically significant.

## Results

### Demographic and clinical characteristics

**Figure 1:**
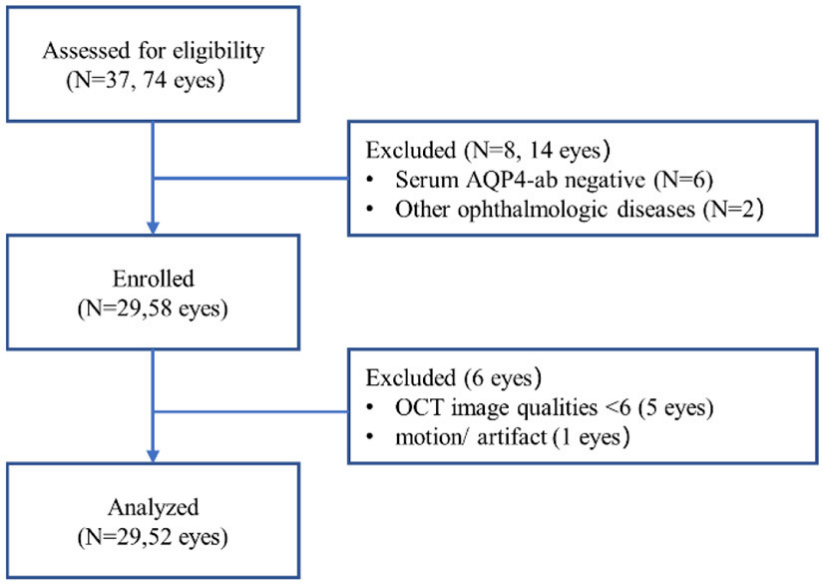
Flow chart showing subject recruitment.

**Figure 2:**
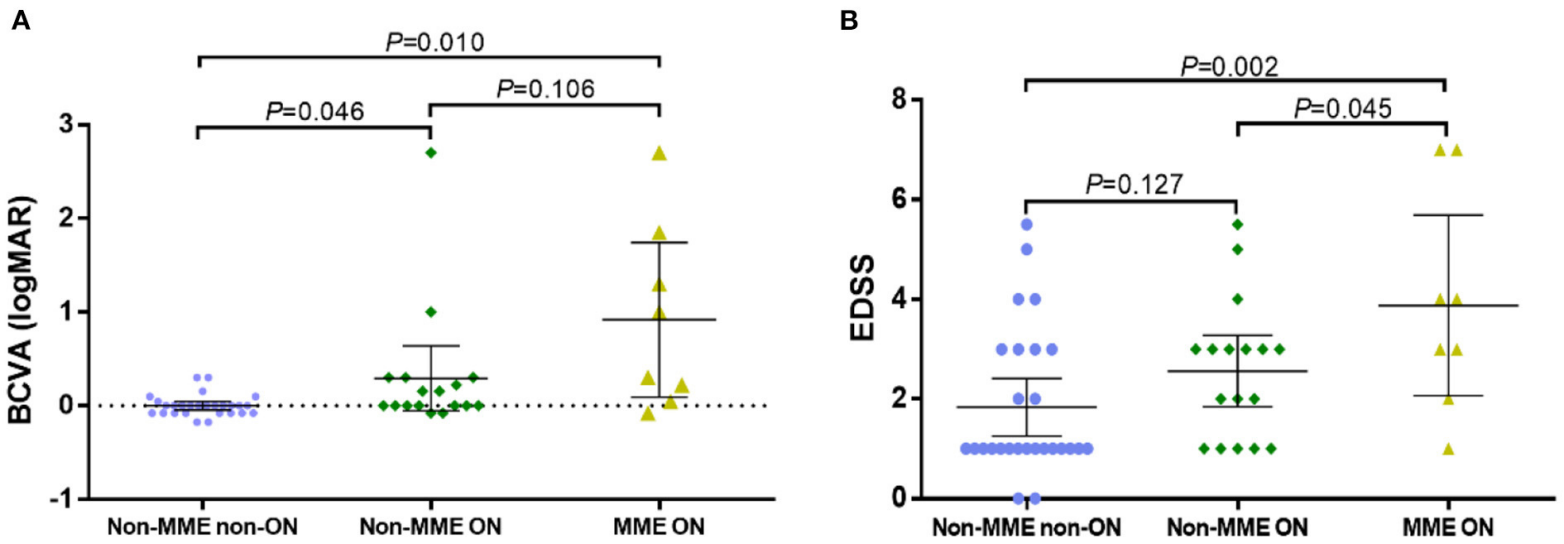
Best corrected visual acuity (BCVA) and the score of Expanded Disability Status Scale (EDSS) in NMOSD patients with non-MME non-ON, non-MME ON and MME ON eyes. ON in NMOSD is associated with worse visual impairment **(A)**, and MME in NMOSD is related with greater disability (EDSS) **(B)**. BCVA, best corrected visual acuity; EDSS, Expanded Disability Status Scale; MME, microcystic macular edema; ON, optic neuritis.

A total of 52 eyes from 29 patients were included (Figure 1). We identified 8 eyes from 7 patients, namely, 24.1% of all patients or 15.4% of eyes, with MME (MME was bilateral in 2 patients). All eyes with MME had a history of ON. The BCVA (logMAR) of the NMOSD MME ON group and the non-MME ON group was significantly worse than in the NMOSD non-MME non-ON group (*P* < 0.05 each). There was no significant difference between non-MME ON and MME ON group (Figure 2A). Patients with MME and ON eyes had worse disability (as measured by median EDSS score) than those in the non-MME ON group (*P* = 0.045) and non-MME non-ON group (*P* = 0.002). However, the EDSS score of the non-MME ON group was not higher than that of non-MME non-ON group (*P* = 0.127) (Figure 2B). Other demographic and clinical characteristics of the participants and the basic ophthalmic information were listed in Table 1.

**Table 1:** Population, ocular demographics, and clinical summary.

	**NMOSD non-MME non-ON**	**NMOSD non-MME ON**	**NMOSD MME ON**	***P*-value**
**Population characteristics**				
Number of participants	11	11	7	NA
Gender (Male/Female)	0/11	0/11	0/7	NA
Age, years (Mean ± SD)	54.23 ± 8.30	43.58 ± 16.60	42.73 ± 14.01	0.118
BMI (Mean ± SD)	23.54 ± 3.53	23.01 ± 2.91	21.51 ± 2.62	0.454
**Ocular characteristics**				
Number of eyes	27	17	8	NA
AL, mm (Mean ± SD)	23.28 ± 1.09	23.65 ± 0.74	23.51 ± 0.88	0.115
SE, D (Mean ± SD)	−0.55 ± 2.38	−1.80 ± 2.79	−1.27 ± 2.3	0.084
IOP, mmHg (Mean ± SD)	13.43 ± 3.1	13.22 ± 2.43	13.48 ± 1.53	0.804

NMOSD, neuromyelitis optica spectrum disorder; MME, microcystic macular edema; ON, optic neuritis; BMI, body mass index; AL, axial length; SE, spherical equivalent; IOP, intraocular pressure; NA, not applicable.

### Macular vessel density

**Figure 3:**
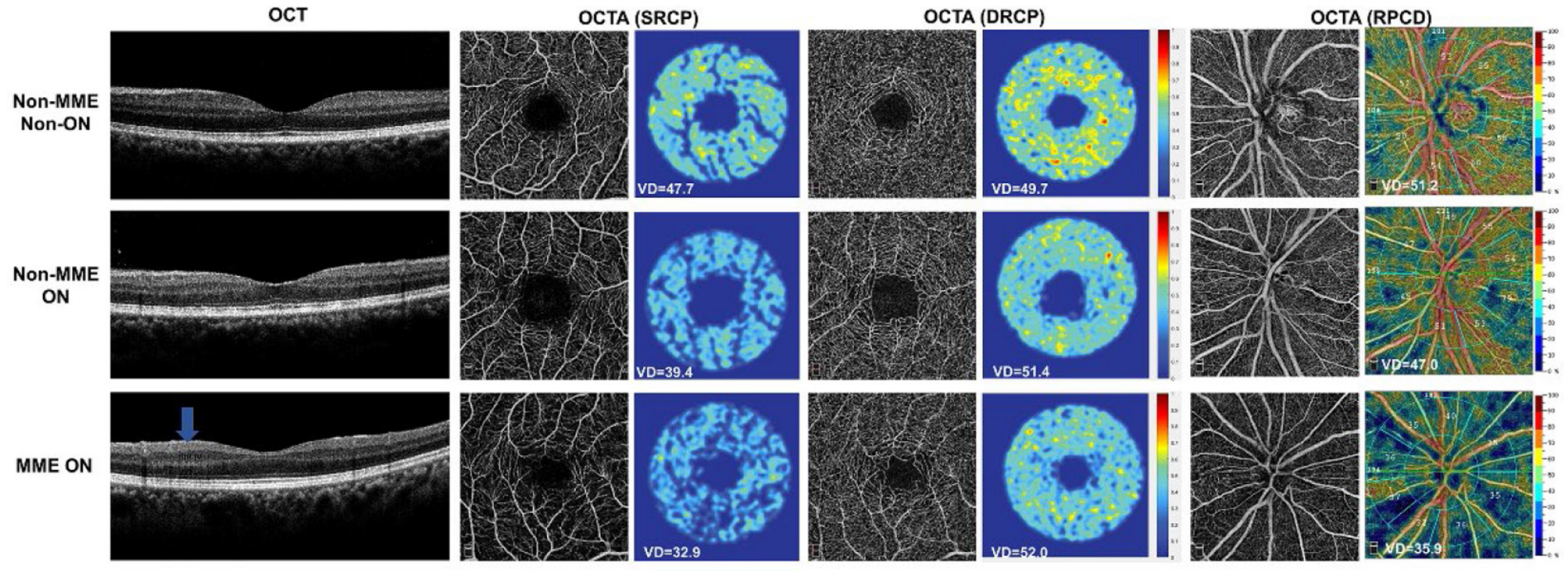
Representative OCT and OCTA images and retinal capillary perfusion maps of non-MME non-ON, non-MME ON, and MME ON eyes. The OCT image in the third row shows that that microcystic macular edema located in the inner nuclear layer (blue arrow). OCT, optical coherence tomography; OCTA, optical coherence tomography angiography; SRCP, superficial retinal capillary plexus; DRCP, deep retinal capillary plexus; RPCD, radial peripapillary capillary density; MME, microcystic macular edema; ON, optic neuritis.

**Figure 4:**
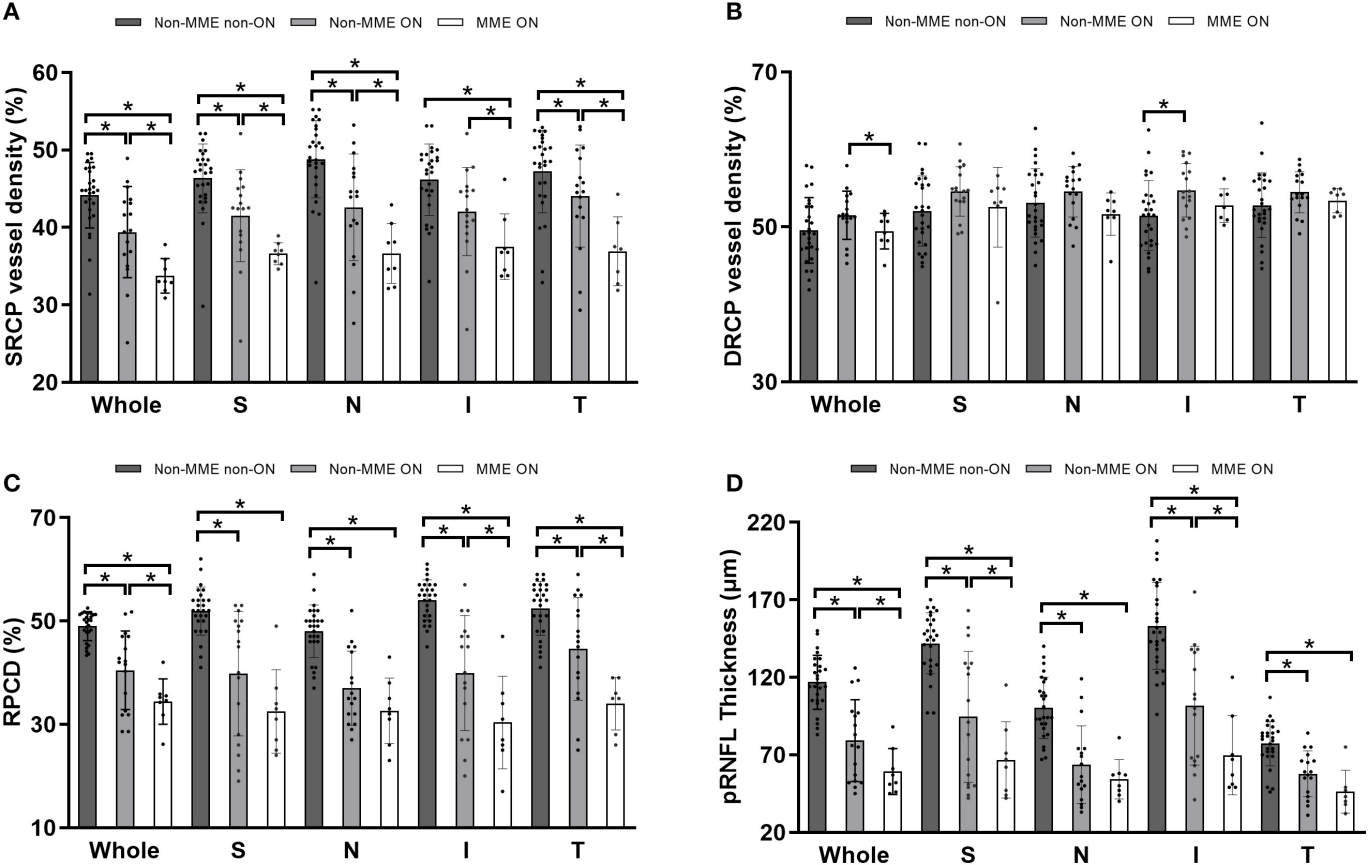
**(A,B)** Comparison of superficial retinal capillary plexus (SRCP) and deep retinal capillary plexus (DRCP) vessel density among groups of non-MME non-ON, non-MME ON, and MME ON eyes. The vessel density in SRCP is significantly lower in eyes with MME ON than in eyes with non-MME ON and non-MME non-ON in the whole macular image and in all grid sections. The vessel density in DRCP is also similar among the three groups. **(C,D)** Comparison of radial peripapillary capillary density (RPCD) and peripapillary retinal nerve fiber layer (pRNFL) thickness among three groups. *P < 0.05. SRCP, superficial retinal capillary plexus; DRCP, deep retinal capillary plexus; RPCD, radial peripapillary capillary density; pRNFL, peripapillary retinal nerve fiber layer; MME, microcystic macular edema; ON, optic neuritis; S, superior; N, nasal; I, inferior; T, temporal.

Representative OCT and OCTA images of the three groups were shown in Figure 3. After adjusting for age and for in-participant inter-eye correlations by the GEE model, the SRCP vessel density was found to be dramatically lower within the NMOSD MME ON group in both the whole macular image and in all grid sections, as compared with that of the non-MME non-ON group (*P* < 0.001), and when compared with the non-MME ON group (*P* < 0.001) (Figure 4A, Table 2). Statistically significant differences were also found between the non-MME non-ON group and the non-MME ON group (*P* = 0.001). Nevertheless, there was almost no significant difference in DRCP vessel density among three groups in both the whole macular image and in all grid sections (Figure 4B, Table 2).

**Table 2:** Comparison of OCT and OCTA parameters among the NMOSD non-MME non-ON, non-MME ON, and MME ON groups.

	**Mean** ±**SD**			**95% Confidence Intervals**			***P*** **-value**		
	**NMOSD**	**NMOSD**	**NMOSD**	**NMOSD**	**NMOSD**	**NMOSD**	**G1 vs. G2**	**G1 vs. G3**	**G2 vs. G3**
	**non-MME**	**non-MME**	**MME**	**non-MME**	**non-MME**	**MME**	
	**non-ON (G1)**	**ON (G2)**	**ON (G3)**	**non-ON (G1)**	**ON (G2)**	**ON (G3)**		
SRCP-whole	44.14 ± 4.23	39.39 ± 5.88	33.75 ± 2.24	42.47–45.82	36.37–42.42	31.88–35.62	0.001	<0.001	<0.001
SRCP-S	48.81 ± 4.98	42.59 ± 6.87	36.65 ± 3.88	46.84–50.78	39.06–46.13	33.41–39.89	0.001	<0.001	0.002
SRCP-N	46.18 ± 4.61	42.04 ± 5.71	37.51 ± 4.24	44.35–48.00	39.11–44.98	33.96–41.06	0.005	<0.001	0.015
SRCP-I	47.20 ± 5.31	44.05 ± 6.60	36.93 ± 4.44	45.10–49.30	40.66–47.45	32.82–41.04	0.050	<0.001	0.001
SRCP-T	46.34 ± 4.45	41.52 ± 5.93	36.60 ± 1.41	44.58–48.10	38.47–44.57	35.42–37.78	0.002	<0.001	0.001
DRCP-whole	49.56 ± 4.24	51.49 ± 3.13	49.45 ± 2.29	47.88–51.23	49.89–53.10	47.54–51.36	0.143	0.659	0.026
DRCP-S	52.05 ± 4.54	54.57 ± 3.18	52.54 ± 5.15	50.26–53.85	52.94–56.20	48.24–56.84	0.051	0.897	0.244
DRCP-N	53.10 ± 4.41	54.54 ± 3.24	51.65 ± 2.74	51.36–54.84	52.87–56.20	49.36–53.94	0.381	0.179	0.008
DRCP-I	51.46 ± 4.52	54.72 ± 3.44	52.76 ± 2.17	49.67–53.25	52.95–56.49	50.75–54.76	0.013	0.311	0.060
DRCP-T	52.79 ± 4.18	54.47 ± 2.66	53.39 ± 1.57	51.14–54.45	53.10–55.84	52.07–54.70	0.147	0.776	0.164
RPCD-whole	49.01 ± 2.80	40.46 ± 7.61	34.44 ± 4.41	47.91–50.12	36.55–44.37	30.75–38.12	<0.001	<0.001	0.006
RPCD-S	51.93 ± 4.63	39.82 ± 12.05	32.50 ± 8.07	50.09–53.76	33.63–46.02	25.75–39.25	<0.001	<0.001	0.064
RPCD-N	48.04 ± 5.07	37.00 ± 7.15	32.63 ± 6.35	46.03–50.04	33.32–40.68	27.32–37.93	<0.001	<0.001	0.083
RPCD-I	54.07 ± 3.92	39.94 ± 11.15	30.38 ± 8.94	52.52–55.63	34.00–45.88	22.90–37.85	<0.001	<0.001	0.017
RPCD-T	52.44 ± 5.22	44.65 ± 9.95	34.00 ± 5.10	50.38–54.51	39.53–49.76	29.28–38.72	0.001	<0.001	<0.001
pRNFL-whole	116.85 ± 17.36	79.18 ± 26.40	59.25 ± 14.89	109.99–123.72	65.60–92.75	46.80–71.70	<0.001	<0.001	0.014
mRNFL-whole	29.17 ± 4.03	21.67 ± 5.55	18.46 ± 5.23	27.57–30.76	18.82–24.53	14.09–22.83	<0.001	<0.001	0.132
GCIPL-whole	68.71 ± 6.85	53.21 ± 10.81	48.09 ± 11.02	66.00–71.42	47.65–58.77	38.87–57.30	<0.001	<0.001	0.197

OCT, optical coherence tomography; OCTA, optical coherence tomography angiography; NMOSD, neuromyelitis optica spectrum disorder; MME, microcystic macular edema; ON, optic neuritis; SRCP, superficial retinal capillary plexus; DRCP, deep retinal capillary plexus; RPCD, radial peripapillary capillary density; pRNFL, peripapillary retinal nerve fiber layer; mRNFL, macular retinal nerve fiber layer; GCL-IPL, ganglion cell layer plus inner plexiform layer; S, superior; N, nasal; I, inferior; T, temporal.

### Peripapillary vessel density and pRNFL thickness

Similar to the tendencies observed in SRCP vessel density, the differences of RPCD in the ONH whole image and in the inferior and temporal section were statistically significant among the groups (Figure 4C, Table 2, all *P* < 0.05). Compared to the non-MME non-ON group, the non-MME ON eyes and the MME ON eyes had remarkably thinner pRNFL thickness in the ONH whole image and in each grid section (all *P* < 0.001). The pRNFL thickness of the MME ON group was significantly lower than in the non-MME ON group in the whole ONH image (*P* = 0.014), in the superior section (*P* = 0.035), and inferior section (*P* = 0.015) (Figure 4D, Table 2, and Supplementary Table 1).

### mRNFL and GCL-IPL thickness

**Figure 5:**
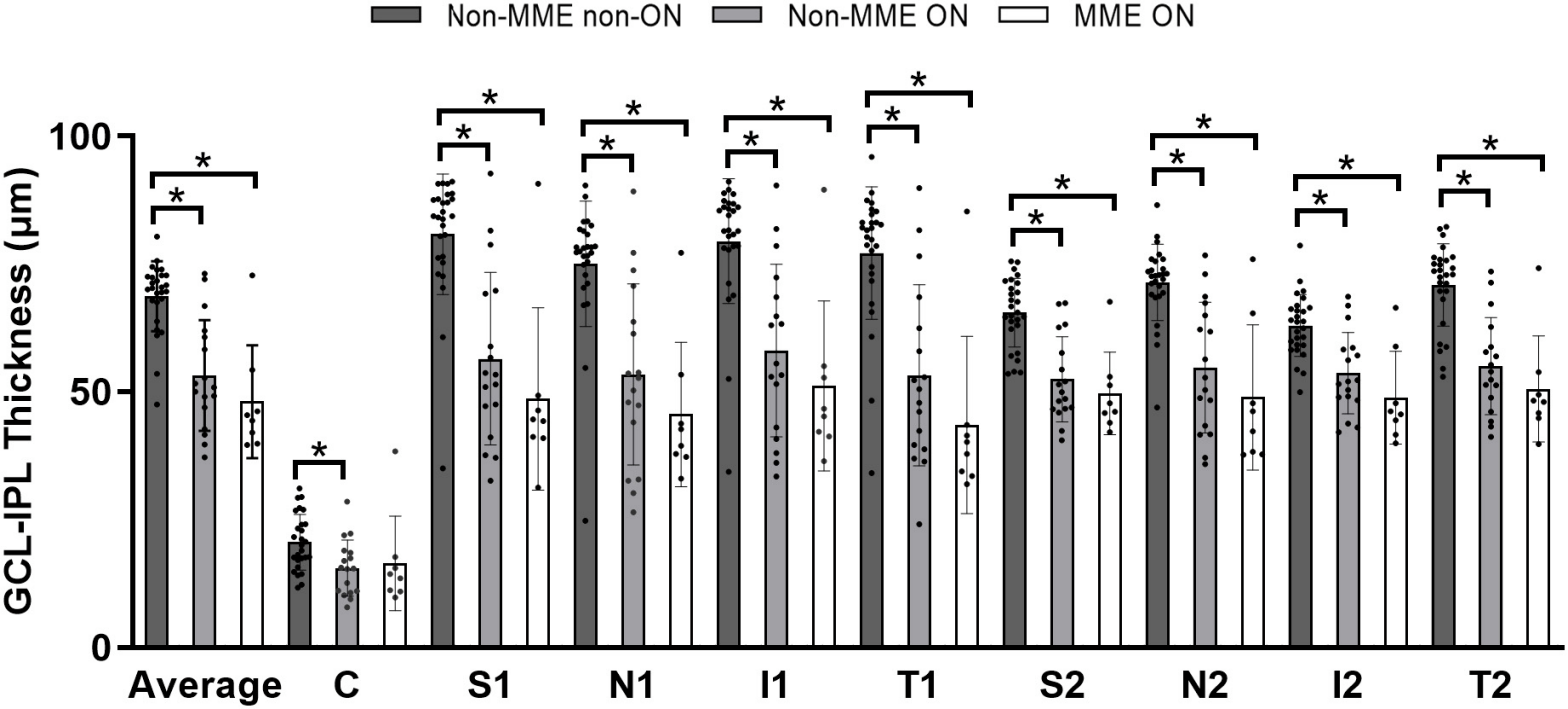
Comparison of ganglion cell layer plus inner plexiform layer (GCL-IPL) thickness among three groups. *P < 0.05. GCL-IPL, ganglion cell layer plus inner plexiform layer; MME, microcystic macular edema; ON, optic neuritis; C, center; S, superior; N, nasal; I, inferior; T, temporal.

There were significant differences between the NMOSD non-MME non-ON and the non-MME ON groups in the thickness of the mRNFL and GCL-IPL in the whole macular image and almost all the sections (*P* < 0.05, expect for the central, T1 and T2 section of the mRNFL) (Figure 5, Table 2, Supplementary Figure 1 and Supplementary Table 1). However, the mRNFL and GCL-IPL thickness were comparable between the non-MME ON group and the MME ON group.

### Correlation between OCT/OCTA parameters and outcomes of visual acuity and disability

We analyzed the correlation of macular and peripapillary vessel density and thickness with BCVA and EDSS values (Table 3). Among the microvascular parameters, the SRCP vessel density and RPCD were negatively associated with BCVA (logMAR) and the EDSS score. In terms of microstructure parameters, the pRNFL, mRNFL and GCL-IPL thickness were found to be negatively correlated with BCVA and the EDSS score.

**Table 3:** The correlation between on OCT and OCTA parameters and clinical endpoints.

**Variables**	**SRCP VD**		**DRCP VD**		**RPCD**		**pRNFL**		**mRNFL**		**GCL-IPL**	
	**B**	***P***	**B**	***P***	**B**	***P***	**B**	***P***	**B**	***P***	**B**	***P***
BCVA (logMAR)	−0.048	<0.001	−0.014	0.556	−0.046	<0.001	−0.010	<0.001	−0.040	0.002	−0.026	<0.001
EDSS	−0.095	0.011	−0.057	0.375	−0.075	0.013	−0.015	0.046	−0.083	0.021	−0.047	0.013

OCT, optical coherence tomography; OCTA, optical coherence tomography angiography; EDSS, Expanded Disability Status Scale; BCVA, best corrected visual acuity; SRCP, superficial retinal capillary plexus; DRCP, deep retinal capillary plexus; VD, vessel density; RPCD, radial peripapillary capillary density; pRNFL, peripapillary retinal nerve fiber layer; mRNFL, macular retinal nerve fiber layer; GCL-IPL, ganglion cell layer plus inner plexiform layer; B, regression coefficient in generalized estimating equation models.

## Discussion

In this study, patients with MME underwent worse visual impairment and more severe disease disability relative to NMOSD patients without MME. Using OCTA with quantification, we found significant abnormalities of the microvascular density in both the macular superficial retinal layer and the peripapillary area in MME eyes. Also, microvascular and structural parameters were correlated to the extent of visual impairment and disability. To the best of our knowledge, this is the first study, using OCTA, to investigate the change of retinal vessel density in NMOSD with MME individuals.

There are many hypotheses about the mechanism of MME, including vitreomacular traction, immune response of Müller cells, and/or trans-synaptic loss of cells (16, 17). Previous studies have mainly focused on the relationship between MME and damage of blood-retina barriers, with little attention paid to intraocular microvasculature changes such as within retinal and peripapillary circulation. A recent study found that the most common location of MME is the nasal and temporal quadrants around the macula, that is, the watershed of the terminal arteries of the choroidal vascular system. These areas have relatively poor blood flow and are most prone to hypoxia and ischemia (18). Eyes showing MME on SD-OCT in the area of edema exhibited diffuse leakage in FA and thus can be considered direct evidence of the destruction of the blood-retinal barrier (10, 19). Observing this, one could surmise the blood-retinal barrier breakdown may occur concurrently with blood-brain barrier breakdown in NMOSD (7). This finding, together with the results in our study, suggests that retinal microvascular perfusion may be involved in the development of MME.

The application of OCTA facilitates our exploration of the pathological mechanism of various diseases in a non-invasive way, such as diabetic retinopathy, glaucoma, and high myopia (14, 20). Our findings of sparse retinal macular and peripapillary microvasculature in patients with NMOSD and MME indicated that decreased microvascular perfusion may serve as a mark of progressive course of NMOSD and was related to clinical endpoints, i.e., visual acuity and disability.

As found within the study, the decrease of macular SRCP vessel density and RPCD were significantly correlated with the existence of MME (Figures 4A,C, Table 2). The SRCP supplies the mRNFL and GCIPL layers which showed no significant difference between non-MME ON and MME ON groups. However, both mRNFL and GCIPL seem to be numerically lower in MME ON eyes. It is possible that reduced SRCP in MME eyes reflect endothelial injury as an effect of prior inflammation or serve as an early sign of neurodegeneration. Another potential explanation for our findings is that vascular changes in NMOSD MME eyes are possible contributors of MME in more advanced courses of NMOSD. ON destroys the microvascular system around the optic nerve, and MME seems to contribute to a second blow in visual outcome. Finally, regardless of whether vascular abnormalities in MME are a primary factor in the pathophysiology of the disease, our results suggest that future studies should take microvasculature into consideration. According to a recent study, OCTA might be a useful biomarker in differentiating NMOSD from MS (21). And as this route has the potential to provide information on the combined effects of neurodegeneration and vasculopathy, it also makes OCTA an attractive tool in the investigation of NMOSD.

Our findings are consistent with previous reports of an association of the occurrence of ON with mRNFL and GCL-IPL thinning in NMOSD (6, 22–24). That being considered, the relationship between MME and neurodegeneration is the primary interest in the current study. The pRNFL is measured in ring scans circling the optic nerve head virtually, where all axons exit the eye (25), thus the thickness of pRNFL may serve as a sigh of early neurodegeneration. In the current study, pRNFL thickness of MME ON group was significantly lower than that of non-MME ON group, and the mRNFL and GCL-IPL thickness were also numerically decreased although not statistically significant, suggesting that MME may correlated with neurodegeneration. In addition, the RPCD also showed a dramatical decrease in MME ON group compared with non-MME ON group, showing that the microvasculature in optical area was also damaged. A more definitive understanding of the relationship between MME and neurodegeneration needs to be established in future research.

The combination concerning history of ON observed in the analysis is another novel finding in our study. Interestingly, all eyes with MME had been attacked by ON, which is consistent with several previous results (23). In patients without MME, the two groups of patients with or without ON showed statistical differences in BCVA, while in patients with ON, whether there was MME or not, did not cause a difference in BCVA (Figure 2A). This result implied that the occurrence of ON may be more relevant explanation for visual impairment, whereas MME may serve as a sensitive biomarker of disability since the EDSS differences were found in non-MME ON and MME ON group. However, regardless of the visual impairment or the degree of disability, the MME ON group suffered more serious clinical damage, which indicated the occurrence of ON and MME might be a biomarker of a more severe clinical course.

There were several limitations in our study. First, the sample size was relatively small, which limited statistical power. Second, FA was not included in the current research. Future studies would benefit from the addition of FA, since leakage of fluorescein would be direct evidence of blood-retina barrier damage. Third, the radial model we used scanned at intervals of 20 degrees, which may result in the omission of smaller MME.

In conclusion, we demonstrated that patients with NMOSD and MME had a significant decrease in retinal perfusion, including more pronounced macular and peripapillary microvascular changes compared to patients without MME. NMOSD MME patients also exhibited neurodegenerative changes found mainly in the peripapillary area. These retinal vascular and structural parameters were found to be relevant to visual impairment and disability. The results also suggest that OCT and OCTA measurement of retinal structure may be useful in evaluating retinal involvement and predicting the degree of clinical endpoints damage in NMOSD.

## Data availability statement

The raw data supporting the conclusions of this article will be made available by the authors, without undue reservation.

## Ethics statement

The studies involving human participants were reviewed and approved by Eye Hospital of the Wenzhou Medical University. Num: KYK [2017] No. 6. The patients/participants provided their written informed consent to participate in this study.

## Author contributions

JL, YC, YZ, ZH, HY, CS, MS, and FL: study concept and design. JL, YC, YZ, HY, and CS: acquisition, analysis, and interpretation of data. JL and MS: drafting of the manuscript. JL: statistical analysis. ZH: administrative and technical or material support. All authors: critical revision of the manuscript for important intellectual content. All authors contributed to the article and approved the submitted version.

## Funding

This work was supported by the National Key R&D Program of China (No. 2020YFC2008200) and Natural Science Foundation of China (Grant No. 82171016).

## Conflict of interest

The authors declare that the research was conducted in the absence of any commercial or financial relationships that could be construed as a potential conflict of interest.

## Publisher's note

All claims expressed in this article are solely those of the authors and do not necessarily represent those of their affiliated organizations, or those of the publisher, the editors and the reviewers. Any product that may be evaluated in this article, or claim that may be made by its manufacturer, is not guaranteed or endorsed by the publisher.

## Supplementary material

Supplementary Figure 1 Comparison of macular retinal nerve fiber layer (mRNFL) thickness among three groups. ^*^*P* < 0.05. mRNFL, macular retinal nerve fiber layer; MME, microcystic macular edema; ON, optic neuritis; C, center; S, superior; N, nasal; I, inferior; T, temporal.

Supplementary Table 1 Comparison of OCT and OCTA parameters among the NMOSD non-MME non-ON, non-MME ON, and MME ON groups.
