# Supplementary figures and images for: Visual function and disability are associated with microcystic macular edema, macular and peripapillary vessel density in patients with neuromyelitis optica spectrum disorder
Source: Front Neurol. 2022 Nov 14;13:1019959. doi: 10.3389/fneur.2022.1019959 (PMC9702058; doi:10.3389/fneur.2022.1019959)

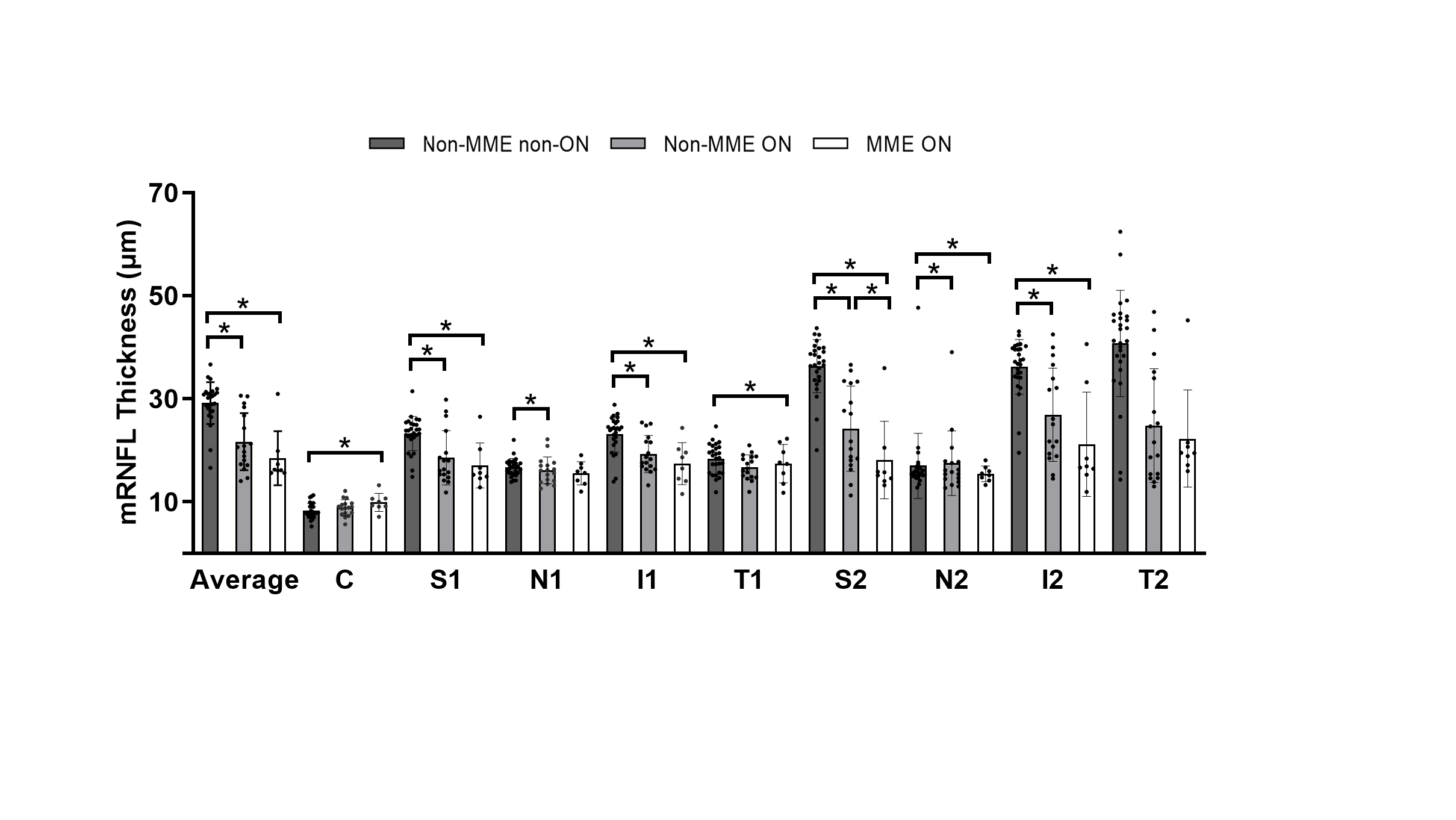

Supplement: Supplementary Figure 1 — Comparison of macular retinal nerve fiber layer (mRNFL) thickness among three groups. *P < 0.05. mRNFL, macular retinal nerve fiber layer; MME, microcystic macular edema; ON, optic neuritis; C, center; S, superior; N, nasal; I, inferior; T, temporal. [file Image_1.tif]
