# Supplementary material for: Visual function and disability are associated with microcystic macular edema, macular and peripapillary vessel density in patients with neuromyelitis optica spectrum disorder
Source: Front Neurol. 2022 Nov 14;13:1019959. doi: 10.3389/fneur.2022.1019959 (PMC9702058; doi:10.3389/fneur.2022.1019959)
Supplement: Supplementary Table 1 — Comparison of OCT and OCTA parameters among the NMOSD non-MME non-ON, non-MME ON, and MME ON groups. [file Table_1.docx]

Table S1. Comparison of OCT and OCTA Parameters Among the NMOSD non-MME non-ON, non-MME ON, and MME ON Groups

|  | Mean ± SD | | |  | 95% Confidence Intervals | | | P value | | |
| --- | --- | --- | --- | --- | --- | --- | --- | --- | --- | --- |
|  | NMOSD non-MME non-ON (G1) | NMOSD non-MME ON (G2) | NMOSD MME ON (G3) |  | NMOSD non-MME non-ON (G1) | NMOSD non-MME ON (G2) | NMOSD MME ON (G3) | G1 vs G2 | G1 vs G3 | G2 vs G3 |
| pRNFL-S | 141.93±19.70 | 94.53±42.24 | 66.75±24.67 |  | 134.13 to 149.72 | 72.81 to 116.25 | 46.13 to 87.37 | <0.001 | <0.001 | 0.035 |
| pRNFL-N | 100.33±19.80 | 63.65±25.07 | 54.25±12.67 |  | 92.50 to 108.17 | 50.76 to 76.54 | 43.66 to 64.84 | <0.001 | <0.001 | 0.101 |
| pRNFL-I | 153.26±27.97 | 101.75±38.33 | 69.75±25.48 |  | 142.20 to 164.32 | 81.32 to 122.18 | 48.45 to 91.05 | <0.001 | <0.001 | 0.015 |
| pRNFL-T | 77.19±14.34 | 57.82±14.70 | 46.29±13.88 |  | 71.51 to 82.86 | 50.26 to 65.38 | 33.45 to 59.12 | <0.001 | <0.001 | 0.093 |
|  |  |  |  |  |  |  |  |  |  |  |
| mRNFL-C | 8.23±1.47 | 8.82±1.59 | 9.87±1.75 |  | 7.65 to 8.81 | 8.01 to 9.64 | 8.41 to 11.33 | 0.232 | 0.029 | 0.073 |
| mRNFL-S1 | 23.23±3.34 | 18.54±5.28 | 17.06±4.38 |  | 21.91 to 24.56 | 15.82 to 21.25 | 13.40 to 20.72 | 0.002 | <0.001 | 0.418 |
| mRNFL-N1 | 18.30±2.93 | 16.71±2.39 | 17.42±3.72 |  | 17.14 to 19.46 | 15.48 to 17.94 | 14.31 to 20.52 | 0.044 | 0.400 | 0.576 |
| mRNFL-I1 | 23.18±3.52 | 19.30±3.61 | 17.42±4.09 |  | 21.78 to 24.57 | 17.45 to 21.16 | 14.00 to 20.84 | 0.001 | <0.001 | 0.183 |
| mRNFL-T1 | 16.65±1.74 | 16.08±2.59 | 15.53±2.26 |  | 15.96 to 17.34 | 14.74 to 17.41 | 13.64 to 17.42 | 0.277 | 0.047 | 0.432 |
| mRNFL-S2 | 36.34±5.14 | 24.18±8.29 | 18.13±7.53 |  | 34.31 to 38.38 | 19.92 to 28.44 | 11.83 to 24.42 | <0.001 | <0.001 | 0.049 |
| mRNFL-N2 | 40.74±10.34 | 24.79±11.05 | 22.28±9.44 |  | 36.65 to 44.83 | 19.11 to 30.47 | 14.39 to 30.17 | <0.001 | <0.001 | 0.541 |
| mRNFL-I2 | 36.17±5.31 | 26.89±9.09 | 21.18±10.10 |  | 34.07 to 38.27 | 22.22 to 31.56 | 12.74 to 29.62 | <0.001 | <0.001 | 0.126 |
| mRNFL-T2 | 16.98±6.35 | 17.50±6.25 | 15.44±1.50 |  | 14.47 to 19.49 | 14.29 to 20.71 | 14.19 to 16.69 | 0.631 | 0.147 | 0.170 |
|  |  |  |  |  |  |  |  |  |  |  |
| GCIPL-C | 20.61±5.44 | 15.55±5.47 | 16.43±9.24 |  | 18.45 to 22.76 | 12.74 to 18.36 | 8.71 to 24.16 | 0.001 | 0.157 | 0.793 |
| GCIPL-S1 | 80.85±11.80 | 56.49±16.89 | 48.61±17.83 |  | 76.19 to 85.52 | 47.81 to 65.18 | 33.71 to 63.52 | <0.001 | <0.001 | 0.269 |
| GCIPL-N1 | 77.14±12.94 | 53.24±17.73 | 43.55±17.36 |  | 72.02 to 82.26 | 44.12 to 62.36 | 29.04 to 58.06 | <0.001 | <0.001 | 0.168 |
| GCIPL-I1 | 79.50±12.24 | 58.10±16.86 | 51.16±16.65 |  | 74.66 to 84.34 | 49.43 to 66.77 | 37.24 to 65.08 | <0.001 | <0.001 | 0.268 |
| GCIPL-T1 | 75.08±12.25 | 53.41±17.70 | 45.59±14.10 |  | 70.23 to 79.93 | 44.31 to 62.51 | 33.80 to 57.38 | <0.001 | <0.001 | 0.192 |
| GCIPL-S2 | 65.51±6.68 | 52.46±8.33 | 49.71±8.07 |  | 62.87 to 68.15 | 48.18 to 56.74 | 42.97 to 56.46 | <0.001 | <0.001 | 0.344 |
| GCIPL-N2 | 70.90±8.05 | 55.07±9.50 | 50.55±10.37 |  | 67.72 to 74.09 | 50.19 to 59.95 | 41.88 to 59.22 | <0.001 | <0.001 | 0.231 |
| GCIPL-I2 | 62.97±6.05 | 53.64±7.96 | 48.87±9.09 |  | 60.58 to 65.37 | 49.55 to 57.74 | 41.27 to 56.47 | <0.001 | <0.001 | 0.104 |
| GCIPL-T2 | 71.40±7.44 | 54.77±12.79 | 48.93±14.22 |  | 68.46 to 74.34 | 48.20 to 61.35 | 37.05 to 60.82 | <0.001 | <0.001 | 0.201 |

OCT, optical coherence tomography; OCTA, optical coherence tomography angiography; NMOSD, neuromyelitis optica spectrum disorder; MME, microcystic macular edema; ON, optic neuritis; pRNFL, peripapillary retinal nerve fiber layer; mRNFL, macular retinal nerve fiber layer; GCL-IPL, ganglion cell layer plus inner plexiform layer; S, superior; N, nasal; I, inferior; T, temporal.
